# Mechanism of Prominent Trimethylamine Oxide (TMAO) Accumulation in Hemodialysis Patients

**DOI:** 10.1371/journal.pone.0143731

**Published:** 2015-12-09

**Authors:** Xin Hai, Veeda Landeras, Mirela A. Dobre, Peter DeOreo, Timothy W. Meyer, Thomas H. Hostetter

**Affiliations:** 1 Department of Medicine, Case Western Reserve University School of Medicine, Cleveland, Ohio, United States of America; 2 Department of Medicine, Stanford University School of Medicine, Palo Alto, California, United States of America; Medical University of Graz, AUSTRIA

## Abstract

Large size, protein binding and intracellular sequestration are well known to limit dialytic removal of compounds. In studying the normal renal and dialytic handling of trimethylamine oxide (TMAO), a molecule associated with cardiovascular disease in the general population, we discovered two largely unrecognized additional limitations to sustained reduction of a solute by chronic hemodialysis. We measured solute levels and handling in subjects on chronic hemodialysis (ESRD, n = 7) and compared these with levels and clearance in normal controls (NLS, n = 6). The ESRD patients had much higher peak predialysis plasma levels of TMAO than NLS (77 ± 26 vs 2±1 μM, mean ± SD, p<0.05). For comparison, predialysis BUN levels in ESRD subjects were 45±11 mg/dl and 15±3 mg/dl in NLS. Thus TMAO levels in ESRD average about 40 fold those in NLS while BUN is 3 fold NLS. However, the fractional reduction of TMAO concentration during dialysis, was in fact greater than that of urea (86±3 vs 74±6%, TMAO vs urea, p < 0.05) and its dialytic clearance while somewhat lower than that of urea was comparable to creatinine’s. Also production rates were similar (533±272 vs 606 ± 220 μ moles/day, ESRD vs NLS, p>0.05). However, TMAO has a volume of distribution about one half that of urea. Also in NLS the urinary clearance of TMAO was high (219±78 ml/min) compared to the urinary urea and creatinine clearances (55±14 and 119±21 ml/min, respectively). Thus, TMAO levels achieve multiples of normal much greater than those of urea due mainly to 1) TMAO’s high clearance by the normal kidney relative to urea and 2) its smaller volume of distribution. Modelling suggests that only much more frequent dialysis would be required to lower levels Thus, additional strategies such as reducing production should be explored. Furthermore, using urea as the sole marker of dialysis adequacy may be misleading since a molecule, TMAO, that is dialyzed readily accumulates to much higher multiples of normal with urea based dialysis prescriptions.

## Introduction

Multiple wastes accumulate with treated ESRD[1]. The urea based prescription for chronic hemodialysis constitutes the prevailing standard of care for people maintained on chronic hemodialysis. However, urea clearance does not reflect the clearance of many other classes of solutes. For example larger molecules such as beta 2 microglobulin, protein bound molecules such as indoxyl sulfate and sequestered solutes such as phosphate and aliphatic amines are well known to be poorly removed with urea targeted therapy[2]. In analyzing the handling of TMAO, a recently described risk factor for cardiovascular disease in the general population, we found that its small volume of distribution and higher native kidney clearance relative to those of urea lead to very high accumulation of TMAO in people receiving good dialysis as judged by urea removal[3].

## Methods

### Subjects

Studies were conducted in seven adult subjects with ESRD stably maintained on thrice weekly hemodialysis and in six control adult subjects with no known renal disease. The subjects with ESRD were studied during their mid- week dialysis session. The subjects had samples of blood and dialysate obtained at the initiation, conclusion, and at 60 to 90 minutes after starting dialysis (mean at 74 minutes) during the dialysis session. The duration of dialysis ranged 3.5 to 4.17 hrs with a mean = 3.96 and standard deviation of .21 hrs. The subjects were dialyzed with Baxter Xenium XPH 170 (n = 1) or Baxter Xenium XPH 150 (n = 6) dialyzers. Their surfaces areas are 1.7 and 1.5 m^2^, respectively. Their ultrafiltration coefficients are 74 and 67 ml/hr/mmHg, respectively. The mass transfer coefficients are greater than 600 ml/min. Blood flow rates ranged from 250–500 ml/min. Dialysate flows were 800 ml/min. The normal subjects had a blood sample drawn during a 24 hour collection of urine. The blood samples were centrifuged within 10 minutes of being obtained. The study was approved by the Institutional Review Board for Human Investigation of University Hospital Case Medical Center,11100 Euclid Ave, Lakeside Building Room 1400, Cleveland Ohio, 44106. All participants signed an informed consent approved by the above IRB in the manner approved by the IRB.

### Analytic methods

#### Determination of TMAO, choline and betaine by LC-MS/MS

The concentration of TMAO, choline and betaine in urine, plasma, dialysate and ultrafiltrate was determined by LC-MS/MS. The molecular weight of TMAO is 75.22 g/mol. Urine was diluted 10 times with water before sample preparation. 20 μl of sample was added to a 1.5 ml microcentrifuge tube containing 10 μl of internal standard mixture (75 μM TMAO-d9, 50 μM choline-d9 and 50 μM betaine-d9). After briefly mixing, 200 μl of methanol was added to precipitate protein. The mixture was mechanically vortexed for 2 min and centrifuged at 14,000×g for 5 min at 4°C.

Sample supernatant (5 μL) was injected to a Shimadzu Prominence LC system (Kyoto, Japan) coupled to an API 4000 triple quadrupole mass spectrometer (AB Sciex, Canada). Analytes were separated on a phenomenex Luna Silica column (150 mm x 2.1 mm, 3 μm particle size) protected by a guard column (4 mm x 2.1 mm silica filter) at room temperature. The mobile phase consisted of 90% methanol containing 10 mM ammonium formate and 0.2% formic acid (v/v) and 10% of 10 mM ammonium formate containing 0.2% formic acid (v/v) and delivered isocratically at a flow rate of 0.2 mL/min. The compounds were ionized in the electrospray ionization operated in the positive mode. Ionizing voltage was 5500 V, and ion source temperature was 650°C. Collision gas: 7, curtain gas: 20, GS1: 60, GS2: 50. Total ion current chromatograms were obtained by a mass spectrometer in multiple reaction monitoring mode. The ion pairs used for the qualitative analysis were m/z 76→58 for TMAO, m/z 85→66 for TMAO-d9, m/z 104→60 for choline, m/z 113→69 for choline-d9, m/z 118→ 59 for betaine, m/z 127→68 for betaine-d9. Data was collected and analyzed using Analyst 1.6 software (AB Sciex, Canada).

#### Creatinine and urea measurement

Creatinine and urea was measured by the same LC-MS/MS method with minor modification of sample preparation. The molecular weights for creatinine and urea are 113.12 g/mol and 60.1 g/mol, respectively. Urine sample was diluted 50 times with water. 10 μl of plasma, diluted urine, dialysate or ultrafiltrate was added to a 1.5 ml microcentrifuge tube containing 10 μl of internal standard mixture (50 μM urea-[^13^C_1_, ^15^N_2_] and 1000 μM creatinine-d3). After briefly mixing, 1 ml of methanol was added to precipitate protein. The mixture was mechanically vortexed for 2 min and centrifuged at 14,000×g for 5 min at 4°C. 5 μL supernatant was injected to the LC-MS/MS system. The fragment ions were observed at m/z 114→44 for creatinine, m/z 117→47 for creatinine-d3, m/z 61→44 for urea and m/z 64→46 for urea-[^13^C_1_, ^15^N_2_].

### Calculations

Mean plasma and dialysate level of solutes during the dialysis session were calculated assuming first order kinetics. More frequent sample measurements and other modelling would be needed to completely define the kinetic behavior of TMAO. The total mass of solute removed during dialysis were calculated as the mean dialysate level times the total dialysate flow plus ultrafiltrate. The amount removed was divided by two to allow comparison with 24 hour excretion in normal subjects. Clearance for a solute was calculated as the rate of removal over the dialysis period divided by the mean plasma level. The volume of distribution for a solute was calculated as the quotient of the total amount removed and the change in plasma level from the beginning to the end of the dialysis session. We did not obtain 30–60 minute post dialysis samples

Data are presented as mean ± standard deviations. Statistical comparisons were assessed using Student’s t test for paired and unpaired data as appropriate. Correlations were assessed using the Pearson correlation coefficient.

## Results

Values for solute plasma levels, clearance rates, and excretion rates are summarized in Table 1. Values for creatinine and urea nitrogen were in accord with expectations in normal subjects. The plasma creatinine value of .85 ± .18 mg/dl (mean ± standard deviation) was accounted for by a urinary excretion of 1432 ± 422 mg/day and a urinary clearance of 119 ± 21ml/min. The plasma urea nitrogen of 15 ± 3 mg/dl was higher in relation to the urea nitrogen excretion of 24.0 ±6.0 g/day, reflecting a lower urea clearance due to the well-known tubular reabsorption of urea. The fractional excretion of urea thus averaged .47± .10 in accord with reported values in normal subjects [4]. In contrast, the plasma TMAO of 2 ± 1uM was lower in relation to the TMAO excretion of 533±248 umoles/day. The urinary TMAO clearance of 219 ± 78 ml/min was thus higher than that of creatinine, indicating active secretion of this solute.

**Table 1:** Solute plasma levels, clearance rates, and excretion rates for study participants.

	Plasma Levels			Clearances*			Excretion/Removal**	
Solute	UN***	Creatinine	TMAO	Urea	Creatinine	TMAO	Urea	TMAO
	mg/dl	mg/dl	μM	ml/min			g/day	μmoles/day
Normals (n = 6)	15±3	.85±.18	2±1	55±14	119°±21	219° ^‡^±78	24±6	533 ±272
ESRD (n = 7)	45^†^±11	10.79^†^±3.09	77^†^±26	258±58	174° ^†^±52	165°±72	12^†^±6	606 ±220

Means ± standard deviations

^†^ p < .05 Normal vs. ESRD

^°^ p< .05 Creatinine and TMAO clearances vs urea clearance within each group

^‡^ p< .05 TMAO vs creatinine clearance

*Clearances are urinary clearances for normal and dialytic clearances for ESRD.

**Excretion is 24 hour urinary excretion for normal. For ESRD, removal is the amount removed in dialysate and ultrafiltrate divided by 2 to adjust for the 2 day interdialytic interval.

*** UN is urea nitrogen.

In subjects with ESRD receiving chronic hemodialysis treatments, pre dialysis plasma levels of TMAO were markedly higher than in the normal subjects at 77 ± 26 μM (p < .05) (See Table 1). The production rate in the ESRD subjects was 606 ± 220 μmoles /day, a rate not different than the urinary excretion of the normal subjects (p > 0.5). The clearances during dialysis for urea, creatinine and TMAO were 258 ± 58, 174 ± 52 and 165 ±72 ml/min respectively. The dialytic clearance for urea was significantly higher (p< .05) than those for the other two solutes. Dialytic clearances of TMAO and creatinine were not different (p> 0.5). The volume of distribution for TMAO was 19 ± 6 l, which was significantly less (p < .05) than that for urea at 36 ± 10 l and for creatinine 31 ± 9 l. Urea and creatinine volumes of distribution were not significantly different (p>.05). The plasma reduction ratio for TMAO was greater than those for urea and creatinine e (.86± .03 vs .77± .05 vs .71 ±.06, respectively, all p < .05).

The relative plasma concentrations of urea, creatinine and TMAO in ESRD subjects compared to the mean values in normals were strikingly different. Whereas the urea concentration was only 3.0 ± 1.6 fold the mean of the normal subjects, those for creatinine and TMAO were respectively 12.7 ± 3.6 and 40.0 ±13.1 fold normal (all significantly different). Thus despite similar dialytic clearances for creatinine and TMAO, the predialysis TMAO levels were a much larger multiple of normal than those of creatinine.

Plasma levels of betaine and choline tended to be higher in subjects with ESRD (p = .055 for the betaine comparison and p< .05 for the choline comparison). There were no significant correlations between urea and TMAO production in either normal subjects or those with ESRD. There were no significant correlations between plasma levels of TMAO and either of its precursors, choline or betaine, in either group of subjects. However, these analyses contained relatively modest numbers of subjects.

## Discussion

TMAO accumulates to very high levels in people receiving chronic hemodialysis as measured at the peak midweek predialysis. These peak predialysis levels averaged almost 40 fold that in normal subjects. We did not define its entire week’s profile. However, using a computer model the predicted time averaged concentration of TMAO would be 40 μM in ESRD subjects compared to 2 μM in normal subjects[5]. This occurs despite their having production rates similar to normal subjects. This remarkable degree of elevation of TMAO in ESRD patients is due mainly to two factors that have been poorly recognized as limitations for chronic hemodialysis. First TMAO’s clearance by the normal kidney is about four fold that of urea while its clearance by dialysis is somewhat less than that of urea. The ratio of dialytic clearance to normal clearance is therefore much lower for TMAO than for urea. Second, the volume of distribution is lower for TMAO than for urea, so that the inefficiency resulting from the intermittency of conventional dialysis treatment is larger than for urea.

Solutes cleared by the native kidney at greater rates than urea will accumulate to higher multiples of normal in patients because no solute is cleared by hemodialysis at higher rates than urea[3]. Since many solutes including creatinine have higher renal clearances than urea it is not surprising that they circulate at higher multiples of normal than does urea in ESRD. Compounds with yet higher rates of renal secretion display even higher multiples ofnormal [3]. The renal clearance of TMAO at twice the rate of creatinine indicates that at least one half of its excretion is accomplished by secretion. Thus, its extraordinary elevation above normal values in ESRD is due in part to the failure of hemodialysis to provide clearances of the magnitude achieved by renal secretion.

Small volumes of distribution render intermittent hemodialysis less effective in lowering time averaged and peak concentrations of a solute even though it may be well cleared by the dialysis procedure itself. This may at first seem paradoxical. However, small volumes of distribution lead to higher average solute concentrations for two reasons. First, the dialysis procedure is less efficient since the solute concentration is rapidly lowered because a small volume is being cleared. Consistent with this view TMAO shows a greater reduction ratio than urea despite a higher relative predialysis level. Hence, much of the duration of the treatment is inefficient or “wasted” as blood already with a low level of the solute continues to be cleared with diminishing result. Second, the accumulation of such a solute in the interdialytic period occurs within the smaller volume with a resultant higher level. Eloot and colleagues have noted this effect of volume of distribution on the efficacy of various dialysis schedules for lowering guanidine succinic acid, another compound with a relatively restricted distribution[6].

The small calculated volume of distribution for TMAO may be in part a difference in rates of intracellular to extracellular equilibration compared to urea. However, the similar rates of excretion of TMAO in normal and its removal in ESRD subjects suggest that differences in equilibration are not entirely the explanation.

Using a computer model assuming a single pool, we tested the effects of several extreme alterations of the standard dialysis prescription on time averaged concentrations of a solute with dialytic characteristics of TMAO, namely dialytically cleared as well as urea but with a volume of distribution of only 19 liters as against urea’s 36 liters [7]. We compared those levels to the predicted time averaged urea level with a volume of distribution of 36 liters and the following standard prescription: thrice weekly for 4 hours, a blood flow of 400ml/min, a dialysate flow of 800 ml/min, and a KoA of 800 ml/min. Doubling the time of thrice week dialysis to 8 hours per session yielded a level of the solute with TMAO’s volume of distribution still 23% higher than that of urea. Doubling dialysate flow resulted in a time averaged concentration 46% higher. Increasing frequency to 6 times per week for 2 hours each session achieved a level comparable to that of urea with the standard prescription. Thus, for even well dialyzed solutes but with small volumes of distribution, frequent dialysis would be required to deliver multiples of normal comparable to that of urea with standard therapy. It should be noted that the normal kidneys’ 4 fold higher clearances of TMAO compared to urea would still lead to substantially higher multiples of normal for TMAO and other secreted compounds even with daily hemodialysis.

When we employed the model in the two compartment mode, minimal (less than 5%) differences in the predicted peak TMAO levels were calculated compared to the single pool mode. We acknowledge that we did not obtain post dialysis samples to assess rebound and it is possible that differing rates of intercompartmental equilibration between urea and TMAO may exist but would require such further studies to define. Using the same computer model we tested the effects of varying ultrafiltration rates and also found only modest (less than 10%) differences in predicted peak TMAO levels across the range of ultrafiltration employed.

TMAO like creatinine displayed a dialytic clearance less than that of urea. In the case of creatinine it is well known that creatinine unlike urea does not exit the red cell in the 15 seconds that the blood transits the dialyzer [8]. Indeed, red cells are less permeable to urea and uric acid than to urea and uremic ones are even less permeable to creatinine and uric acid than those from normal subjects thereby further limiting their dialytic removal [9, 10]. Whether other solutes are also selectively hindered in exiting erythrocytes is unknown. Therefore essentially only the plasma compartment of the blood flow is subject to dialysis of creatinine. Judging from its volume of distribution TMAO appears to be largely extracellular and it likely resides mainly in the plasma flowing through the dialyzer leading to a clearance lower than urea but similar to creatinine.

Plasma levels for TMAO have been reported as elevated in ESRD [11]. However, the plasma levels reported for normal subjects were about ten fold those we find and those the Hazen group have found using a similar LC-MS/MS assay[12, 13]. The earlier reports used gas chromatography with mass spectrometry (GC-MS). The analyte actually assayed in the mass spectrometry was volatilized trimethylamine which was thought to be derived from TMAO via a preparatory reduction step performed on the plasma. We suspect that the reductive step generated trimethylamine from multiple sources not just from TMAO and the plasma levels reported in normals were spuriously high with resultant calculations of low renal clearances.

The lack of correlation between urea and TMAO production is consistent with the current view that dietary quaternary ammonia compounds like choline and not protein give rise to TMAO’s direct precursor trimethylamine[12–14]. The lack of correlation between plasma TMAO concentrations and those for choline or betaine is also consistent with the view that choline and betaine are metabolized to trimethylamine in the gut with TMAO produced largely in the liver by oxidation of trimethylamine. However, we acknowledge that the sample size is modest. Studies of mice have demonstrated that increasing the dietary carnitine and choline raises TMAO levels [15, 16]

In summary, TMAO, which has recently been identified as a risk factor for cardiovascular disease in the general population, circulates at very high levels in ESRD subjects receiving chronic hemodialysis[13, 15]. Several physiologic characteristics of TMAO account for this extraordinary elevation. These findings emphasize that urea clearance while a useful marker for dialysis adequacy is not reflective of the dialysis of other potentially more toxic substances[17].

## Supporting Information

S1 File Fig A: Individual data for decay in TMAO level as percentage of baseline (Y axis) versus time on dialysis (X axis) (DOCX)
