# Supplementary material for: Mechanism of Prominent Trimethylamine Oxide (TMAO) Accumulation in Hemodialysis Patients
Source: PLoS One. 2015 Dec 9;10(12):e0143731. doi: 10.1371/journal.pone.0143731 (PMC4674074; doi:10.1371/journal.pone.0143731)
Supplement: S1 File — (DOCX) [file pone.0143731.s001.docx]

Figure A Individual data for decay in TMAO level as a percentage of baseline (Y axis) versus time on dialysis (X axis)
